# Specific requirement for translation initiation factor 4E or its isoform drives plant host susceptibility to *Tobacco etch virus*

**DOI:** 10.1186/1471-2229-14-67

**Published:** 2014-03-19

**Authors:** Joan Estevan, Aramata Maréna, Caroline Callot, Séverine Lacombe, André Moretti, Carole Caranta, Jean-Luc Gallois

**Affiliations:** 1 INRA-UR1052, Genetics and Breeding of Fruits and Vegetables, Dom. St Maurice, CS 60094, Montfavet Cedex F-84143, France

**Keywords:** *Potyvirus*, Translation initiation factor, eIF4E, *Arabidopsis thaliana*, *Capsicum annuum*

## Abstract

**Background:**

In plants, eIF4E translation initiation factors and their eIFiso4E isoforms are essential susceptibility factors for many RNA viruses, including potyviruses. Mutations altering these factors are a major source of resistance to the viruses. The *eIF4E* allelic series is associated with specific resistance spectra in crops such as *Capsicum annum*. Genetic evidence shows that potyviruses have a specific requirement for a given 4E isoform that depends on the host plant. For example, *Tobacco etch virus* (TEV) uses eIF4E1 to infect *Capsicum annuum* but uses eIFiso4E to infect *Arabidopsis thaliana*. Here, we investigated how TEV exploits different translation initiation factor isoforms to infect these two plant species.

**Results:**

A complementation system was set up in *Arabidopsis* to test the restoration of systemic infection by TEV. Using this system, *Arabidopsis* susceptibility to TEV was complemented with a susceptible pepper *eIF4E1* allele but not with a resistant allele. Therefore, in *Arabidopsis*, TEV can use the pepper eIF4E1 instead of the endogenous eIFiso4E isoform so is able to switch between translation initiation factor 4E isoform to infect the same host. Moreover, we show that overexpressing the pepper *eIF4E1* alleles is sufficient to make *Arabidopsis* susceptible to an otherwise incompatible TEV strain. Lastly, we show that the resistant *eIF4E1* allele is similarly overcome by a resistance-breaking TEV strain as in pepper, confirming that this *Arabidopsis* TEV-susceptibility complementation system is allele-specific.

**Conclusion:**

We report here a complementation system in *Arabidopsis* that makes it possible to assess the role of pepper *pvr2-eIF4E* alleles in susceptibility to TEV. Heterologous complementation experiments showed that the idiosyncratic properties of the 4E and iso4E proteins create a major checkpoint for viral infection of different hosts. This system could be used to screen natural or induced eIF4E alleles to find and study alleles of interest for plant breeding.

## Background

Cap-dependent eukaryotic translation is initiated when the cap structure at the 5’ end of the messenger RNA is recognised by the eIF4F protein complex. eIF4F is composed of eIF4E, a small protein that interacts directly with the cap, and eIF4G, a large scaffold protein [1]. Higher plants have another form of eIF4F, the eIFiso4F complex, made up of eIFiso4E and eIFiso4G proteins [2].

Various RNA viruses, especially those belonging to the *Potyvirus* genus, require plant genes encoding these translation initiation factors in order to complete their infectious cycle. *eIF4E*, *eIF4G* and the genes encoding their respective isoforms confer recessive resistance to those viruses [3,4]. *eIFiso4E* was reported to have a role in *Arabidopsis thaliana* resistance to potyviruses *Turnip mosaic virus* (TuMV) and *Tobacco etch virus* (TEV) and concomitantly, eIF4E1 was shown to have a role in *Capsicum annuum* (pepper) resistance to *Potato virus Y* (PVY) and TEV [5-7]. Since then, variability in eIF4E, mainly associated with polymorphisms resulting in Amino Acids (AA) changes within the eIF4E protein, has been revealed as the basis for known resistance alleles in several pathosystems including *Lactuca sativa*/*Lettuce mosaic virus* (LMV) and *Pisum sativum*/*Pea seed-borne mosaic virus* (PSbMV), while eIFiso4E was shown to be involved in the resistance of *Prunus domestica* to *Plum pox virus* (PPV) [8-10]. In *Capsicum annum*, *Pepper veinal mottle virus* and *Chilli veinal mottle virus* are able to use both eIF4E1 and eIFiso4E and consequently, the plant resistance is associated with mutations affecting those two genes [11,12]. Another interesting feature of eIF4E-based resistance/susceptibility is that in the same host different potyviruses specifically recruit different eIF4F isoforms. For example, in *Arabidopsis thaliana* TuMV specifically uses the eIFiso4F complex, whereas the *Clover yellow vein virus* (ClYVV) uses the eIF4F complex [6,13,14].

Potyviruses can affect multiple hosts. The potyviruses LMV, TEV, PPV and ClYVV all affect *Arabidopsis*, although their respective natural hosts would usually be lettuce (*Lactuca sativa*), pepper or tomato (*Solanum lycopersicum*), plum (*Prunus domestica*), and pea (*Pisum sativum)* (Table 1). For each of these viruses, host translation initiation factors 4E are required for infection in both *Arabidopsis* and in crops. PPV relies on the same isoform eIFiso4E for infection of both *Arabidopsis* and plum [10,15] and ClYVV relies on eIF4E in both pea and *Arabidopsis *[13,16]. Interestingly, TEV and LMV use different isoforms depending on which plant species is being infected [5-7,9,14,17,18].

**Table 1:** **Reported 4E isoforms involved in susceptibility to the same potyvirus in crops and in *Arabidopsis***

**Virus**	**Crop**	**Susceptibility 4E in crop**	**Susceptibility 4E in *Arabidopsis***	**References**
*Clover yellow vein virus*	*Pisum sativum*	eIF4E1	eIF4E1	[13,16]
*Plum pox virus*	*Prunus domestica*	eIFiso4E	eIFiso4E	[10,15]
*Tobacco etch virus*	*Capsicum annuum*	eIF4E1	eIFiso4E	[5,7]
*Tobacco etch virus*	*Lycopersicon esculentum*	eIF4E1	eIFiso4E	[7,18]
*Lettuce mosaic virus*	*Lactuva sativa*	eIF4E1	eIFiso4E	[6,9]

It is not completely clear yet why different eIF4E protein isoforms are selected to infect different hosts. In the *Arabidopsis*/TuMV and pepper/TEV-PVY pathosystems, it is known that the eIF4E1 or eIFiso4E initiation factors interact specifically with VPg, a virus-encoded protein that is covalently linked to the 5’ end of the viral genomic RNA in place of a cap structure [17,19,20]. However, the correlation between plant susceptibility to a potyvirus and the eIF4E/VPg interaction does not extend to all pathosystems [21,22]. So it is likely that other factors encoded by either the virus or the host are required to strengthen the interaction between the initiation factors and VPg and to specify which isoform, eIF4E or eIFiso4E, is recruited.

Here we endeavoured to see whether the eIF4E or eIFiso4E proteins alone determine which complex is recruited by a particular potyvirus by analysing the TEV-susceptibility that relies on *eIF4E1* in pepper but on *eIFiso4E* in *Arabidopsis*. We focussed on two pepper *eIF4E1* alleles, *pvr2*^+^ and *pvr2*^*2*^ (hereafter *Ca.eIF4E1-pvr2*^*+*^ and *Ca.eIF4E1-pvr2*^*2*^, respectively) and on two TEV strains with contrasting behaviour towards those alleles, HAT and CAA10. The *Ca.eIF4E1-pvr2*^*+*^ allele makes plants susceptible to both the HAT and CAA10 strains. The Ca.eIF4E1-pvr2^2^ allele confers resistance to the TEV HAT strain, but this resistance is overcome by the TEV CAA10 strain [5,17]. We set up a complementation system in *Arabidopsis thaliana* to test whether pepper eIF4E1 can restore susceptibility to a TEV-resistant *Arabidopsis* genotype. We show that the heterologous expression of a pepper eIF4E1 is sufficient to restore susceptibility in *Arabidopsis* plants devoid of the susceptibility factor eIFiso4E and is sufficient to define the resistance spectrum of the *Arabidopsis* host.

## Results

### The requirement by TEV for a specific 4E isoform is not explained by sequence homology or by interaction with the viral VPg

**Figure 1:**
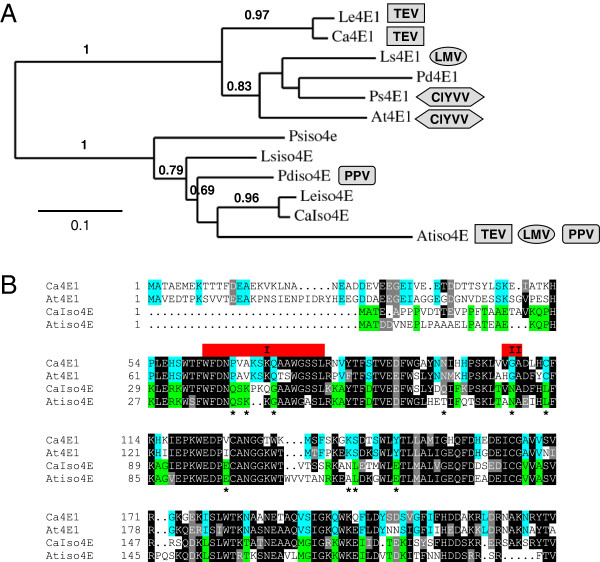
**Resistance to TEV and LMV depends on different isoforms of eIF4E. A**, Phylogenetic tree based on full length eIF4E1 and eIFiso4E protein sequences from *Lycopersicon esculentum* (Le), *Capsicum annum* (Ca), *Lactuva sativa* (Ls), *Pisum sativum* (Ps), *Prunus domestica* (Pd) and *Arabidopsis thaliana* (At). See methods for accession numbers. Bootstrap values over 0.6 supporting the branchpoints are represented. Resistance to potyviruses that have been reported both in *Arabidopsis* and in another plant species, namely TEV, LMV, ClYVV and PPV, are represented by a boxed virus abbreviation next to the 4E protein it has been shown to rely on (See Table 1 for references). **B**, Protein sequence alignment of *Capsicum annuum* and *Arabidopsis thaliana* eIF4E1 and eIFiso4E. Amino acids identical or similar among at least 3 sequences are highlighted in black and grey, respectively. Amino acids common only to either eIF4E1 or eIFiso4E sequences are highlighted in blue and green, respectively. Isoform-specific amino acids as defined by Monzingo et al. [23] are marked by an asterisk. eIF4E1 box I and II, marked in red, are clusters of natural variation involved in resistance to potyviruses, as defined by Robaglia and Caranta [3].

Potyviruses for which 4E-based resistances have been reported both in a crop and in *Arabidopsis* were considered (Table 1). To check that the 4E proteins involved in susceptibility to potyviruses were assigned to the correct isoform group, phylogenies based on their protein sequences were built. Analyses show that 4E proteins belonging to six distantly related angiosperm plant species are correctly divided into eIF4E and eIFiso4E clades (Figure 1A).

We focussed on plant susceptibility to the TEV HAT strain, which involves eIF4E1 in pepper and eIFiso4E in *Arabidopsis*. The sequences of eIF4E1 and eIFiso4E proteins from pepper and *Arabidopsis* were aligned to see whether sequence homologies between Ca.eIF4E1 and At.eIFiso4E could explain why the TEV uses different isoforms depending on the host (Figure 1B). However, the Ca.eIF4E1 protein is much more similar to At.eIF4E1 than to At.eIFiso4E (identity 63.8% and 42.9%, respectively). Overall sequence homologies and the signature residues previously identified [23] both confirm that Ca.eIF4E1 and At.eIF4E1 on one hand and Ca.eIFiso4E and At.eIFiso4E on the other hand are assigned to the correct isoform group. In total, 48 AA are specific to eIF4E1 sequences and 41 AA to eIFiso4E sequences. Among these specific residues, 23 were mutually exclusive. Furthermore, the analysis of several resistant alleles in crops has made it possible to delimit regions I and II in the eIF4E1 protein sequence where AA substitutions involved in resistance to potyviruses tend to cluster [3]. It is possible to delimit region I and II in eIFiso4E because three-dimensional models suggest that eIF4E1 and eIFiso4E adopt a similar structure [24,25]. A higher degree of similarity was expected in regions I and II between Ca.eIF4E1 and At.eIFiso4E, but regions I and II are in fact much more conserved between At.eIF4E1 and Ca.eIF4E1 and between At.eIFiso4e and Ca.eIFIso4E, respectively (Figure 1B). Overall then, protein sequence analyses do not explain why TEV HAT relies on different isoforms to infect *Arabidopsis* and pepper respectively.

**Figure 2:**
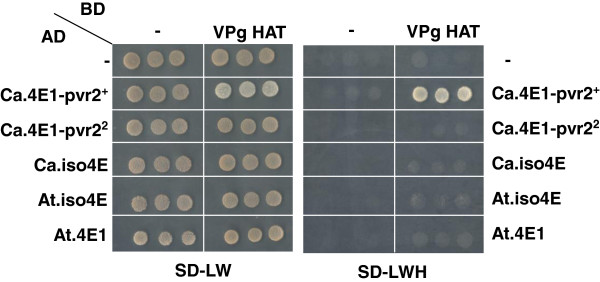
**Arabidopsis TEV-susceptibility protein AteIFiso4E does not interact with TEV VPg in yeast two-hybrid assays.** Yeast transformed with both bait (fused to the Gal4 binding domain, BD) and prey (fused to the Gal4 activation domain, AD) vectors were spotted on selective dropout medium without leucine and tryptophan (SD-LW) as a control and on selective dropout without leucine, tryptophan and histidine (SD-LWH) to check for interaction between both partners. In each case, a control with an empty vector (-) was included to confirm there was no self-activation. All combinations are shown in triplicate.

In pepper and in *Arabidopsis,* physical interaction of eIF4E or eIFiso4E with the potyviral VPg has been shown to correlate with the host susceptibility to the virus. We tested in yeast-two hybrid assays whether differential interaction between 4E initiation factors and the TEV VPg might be responsible for the different isoform requirement between *Arabidopsis* and pepper (Figure 2). As previously reported, we found that the TEV HAT VPg strongly interacts with the susceptible Ca.eIF4E1-pvr2^+^ protein but not with the resistant Ca.eIF4E1-pvr2^2 ^[17]. This differential interaction is restricted to Ca.eIF4E1 proteins as the TEV HAT VPg does not interact with the pepper Ca.eIFiso4E protein. TEV HAT VPg did not interact with either At.eIF4E1 or with At.eIFiso4E, although genetic studies have shown that At.eIFiso4E is required for *Arabidopsis* infection by TEV [6,7,26]. One explanation is that in some species the plant specificity depends on additional factors *in planta* that modulate the interaction between the viral proteins and the 4E initiation factor. Alternatively, additional factors may impair eIF4E1 recruitment by the virus in *Arabidopsis*.

### Setting up a TEV-complementation system in *Arabidopsis*

**Figure 3:**
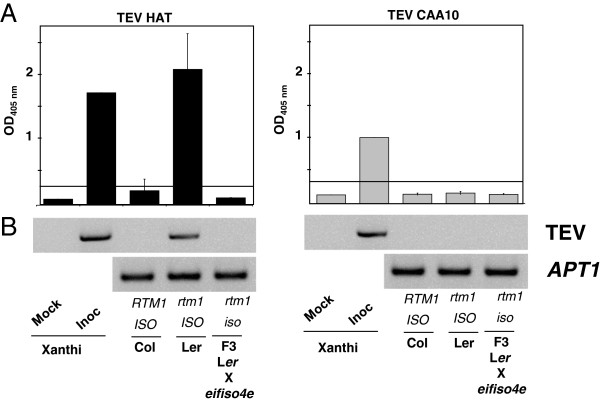
**The *rtm1 eifiso4e *double mutant is resistant to TEV HAT and TEV CAA10 does not infect *Arabidopsis thaliana *L*er *or Col accessions.** One-month-old *Arabidopsis* were manually inoculated with TEV HAT or CAA10 strains and assayed at 24 dpi. Wild type accessions Columbia (*eIFiso4E/eIFiso4E; RTM1/RTM1*) and Landsberg *erecta* (*eIFiso4E/eIFiso4E; rtm1/rtm1*) were compared to the double mutant *eifiso4e/eifiso4e; rtm1/rtm1. Nicotiana tabacum* cv Xanthi non-inoculated (mock) or inoculated (Inoc) plants were included as controls. **A,** Plants were assayed for viral coat protein accumulation by ELISA at 24 dpi. Mean values for 6 independent plants per genotype are shown and error bars represent standard errors. The horizontal black line is the susceptibility threshold. **B,** RT-PCR expression of the TEV VPg gene in systemic leaf tissues. *APT1* is amplified as a constitutive control in *Arabidopsis* plants.

If additional factors affect the interaction of TEV with eIF4E isoforms and are involved in host specificity, expression of a susceptible Ca.eIF4E1 in a TEV-resistant *Arabidopsis* background should not restore susceptibility. We aimed to test whether a pepper eIF4E1 could complement an *Arabidopsis* line lacking its endogenous At.eIFiso4E and hence resistant to TEV. However, in the *Arabidopsis thaliana* Columbia accession, the resistance to TEV triggered by the *eifiso4e* mutation is masked by the presence of *RTM1,* a natural dominant resistance gene that represses the systemic spread of most TEV virus strains including TEV-HAT [27,28]. To circumvent the masking effect of *RTM1* resistance, the Columbia *eifiso4e* line was crossed to Landsberg *erecta* (L*er*), which carries a defective *rtm1* allele. Homozygous *eifiso4e rtm1* double mutants were selected in the F2 population. These plants were allowed to self-fertilise and the TEV susceptibility of the resultant F3 plants was assessed. Columbia (*eIFiso4E/eIFiso4E*; *RTM1/RTM1*), L*er* (*eIFiso4E/eIFiso4E*; *rtm1/rtm1*) and the F3 *eifiso4e rtm1* double mutants (*eifiso4e/eifiso4e*; *rtm1/rtm1*) were challenged with TEV HAT or CAA10. Plant susceptibility was assessed by testing viral gene expression and viral protein expression to check for systemic infection by either virus (Figure 3). As previously reported [28], TEV HAT could systemically infect the L*er* accession but not Columbia. The *eifiso4e rtm1* plants were resistant to TEV HAT suggesting that the *eifiso4e* KO allele is an effective and complete resistance allele to TEV HAT [6]. The TEV CAA10 strain, which overcomes the resistance of the Ca.*eIF4E- pvr2*^*2*^ allele in pepper, was unable to infect either Col or L*er* (Figure 3), suggesting incompatibility or that some other form of resistance is at work.

**Figure 4:**
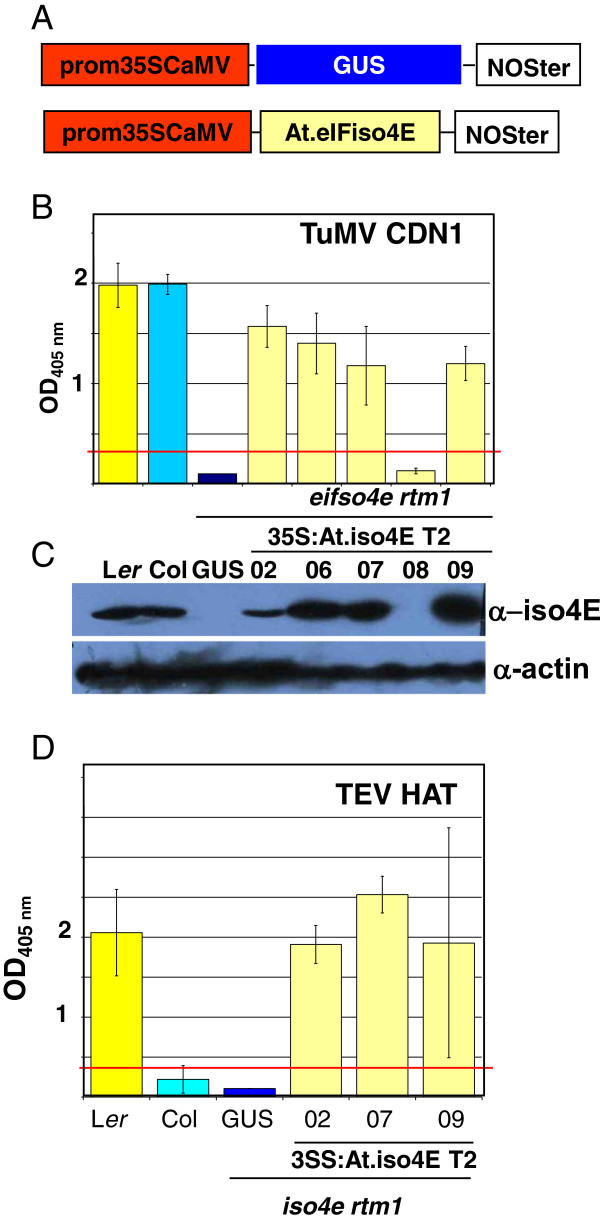
**Homologous complementation of *eifiso4e rtm1 *by AteIFiso4E overexpression restores susceptibility to TuMV and TEV HAT. A**, Schematic representations of the T-DNA constructs inserted in *eifiso4e rtm1 Arabidopsis* plants. **B**, One-month-old *Arabidopsis* plants were inoculated with the TuMV CDN1 strain and assayed for viral coat protein accumulation by DAS-ELISA at 24 dpi. One T2 line transformed with the 35S:GUS construct was tested and five independent T2 lines transformed with 35S:AteIFiso4E. **C**, Western blot analysis of eIFiso4E protein levels in total proteins extracted from 1-month-old leaves. Actin protein levels were assessed as a loading control. **D**, One-month-old *Arabidopsis* plants were inoculated with TEV-HAT and assayed for viral coat protein accumulation by ELISA at 24 dpi.

To validate the complementation system, the *At.eIFiso4E* cDNA was overexpressed in the *eifiso4e rtm1* mutant. As *At.eIFiso4E* mRNA is normally ubiquitously expressed in all *Arabidopsis* tissues, its cDNA was cloned under the control of a 35SCaMV promoter in a binary vector and transformed into *eifiso4e rtm1* plants. As a negative control, a 35S:GUS construct, expressing the reporter gene *uidA,* was transformed in the same background (Figure 4A).

Transgenic plants were first challenged with TuMV CDN1, because the *eifiso4e* KO allele has been described as being resistant to this viral strain [6,20], (Figure 4B). Four out of the five independent 35S:At.eIFiso4E T2 lines tested showed complete susceptibility to TuMV (Figure 4B), showing successful complementation of the *eifiso4e* mutation by overexpressing At.eIFiso4E. In parallel, At.eIFiso4E protein levels were assessed in plant extracts by western blot using a specific polyclonal serum (Figure 4C). A specific band of the expected 21 kDa size was detected in wild-type Col and L*er* plant extracts but was absent in extracts from *eifiso4e rtm1* plants. The four transgenic lines that were susceptible to TuMV were found to accumulate high levels of eIFiso4E. Significantly, expression of the eIFiso4E transgene was not detected in line 08, which remained resistant to TuMV CDN1.

Three of the independent 35S:*AteIFiso4E* lines showing a high level of susceptibility to TuMV were challenged with TEV HAT and were found to be highly susceptible (Figure 4D). These results validated the efficiency of the TEV-susceptibility complementation system.

### Heterologous Ca.eIF4E1 expression in *Arabidopsis* restores susceptibility to TEV HAT

**Figure 5:**
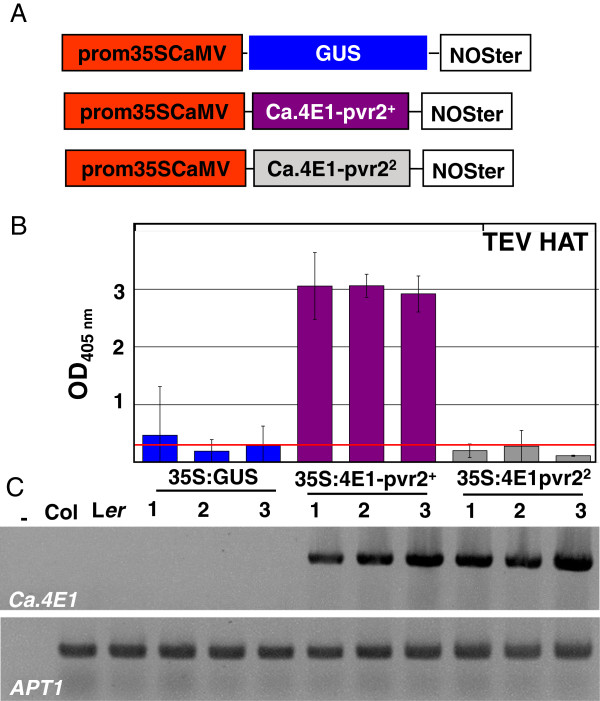
**Heterologous complementation of *eifiso4e rtm1 *with *Capsicum annuum eIF4E1 *alleles trigger susceptibility to TEV HAT in an allele-specific manner. A**, Schematic representations of the T-DNA constructs inserted in *eifiso4e rtm1 Arabidopsis* plants. **B**, One- month-old *Arabidopsis* plants were inoculated with TEV HAT and assayed for viral coat protein accumulation by ELISA at 24 dpi. Results are shown on three independent T2 lines per construct. **C**, RT-PCR on total mRNA extracted from 1-month-old plants show that the *Ca.eIF4E* mRNA is expressed at similar levels in *eifiso4e rtm1* plants transformed with T-DNA harbouring a 35S:*Ca.eIF4E-pvr2*^*+*^ or 35S:*Ca.eIF4E1*-*pvr2*^*2*^ construct. The reference gene *APT1* is amplified as a control.

In order to test whether pepper eIF4E alleles can complement the susceptibility to TEV in *Arabidopsis*, the full length cDNA encoding Ca.eIF4E1*-pvr2*^*+*^ and Ca.eIF4E1-*pvr2*^*2*^ were cloned into a binary vector and transformed into *eifiso4e rtm1 Arabidopsis* plants (Figure 5A). When challenged with TEV HAT, five T2 lines out of six that overexpressed the Ca.eIF4E1-*pvr2*^+^ susceptibility allele accumulated a high level of viral coat protein in systemic tissues, so were highly susceptible to this strain (Figure 5B and data not shown). The pepper eIF4E1 encoded by the *pvr2*^+^ allele can therefore be used by TEV HAT in *Arabidopsis* instead of its heterolog isoform AteIFiso4E. In comparison, the overexpression of the *Ca.eIF4E1*-*pvr2*^*2*^ allele in the same *eifiso4e rtm1* background did not restore susceptibility to TEV HAT in any of the 6 independent lines tested (Figure 5B and data not shown). To ensure that those phenotypes were not due to differences in transgene expression, the levels of *Ca.eIF4E1* mRNA in plant leaves were analysed by RT-PCR (Figure 5C). Similar large amounts of *Ca.eIF4E1* mRNA accumulated in all the lines tested. Even when the *Ca.eIF4E1 pvr2*^*2*^ allele is highly expressed in *eifiso4e rtm1* plants, susceptibility to TEV HAT is not restored*.* Overall, these data show that in *Arabidopsis*, the TEV HAT uses the *Ca.eIF4E1*-*pvr2*^+^ susceptible allele instead of the *At.eIFiso4E*, so is able to swap its 4E isoform requirement within the same host. The susceptibility to TEV could not be restored by the *pvr2*^*2*^ eIF4E1 resistant allele. Hence this TEV-susceptibility complementation system is allele specific.

### Complementation of *Arabidopsis* with heterologous Ca.eIF4E1 generates loss of incompatibility to TEV CAA10

**Figure 6:**
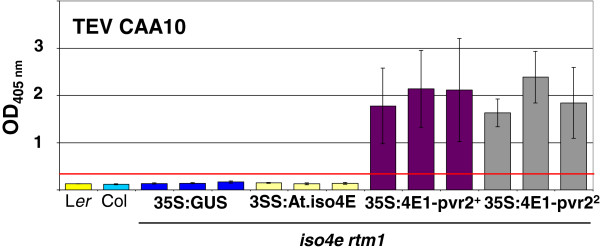
**Heterologous complementation of *eifiso4e rtm1 *with *Capsicum annum eIF4E1 *alleles suppresses incompatibility to TEV CAA10.** One-month-old *Arabidopsis* plants were inoculated with TEV CAA10 strain and assayed for viral coat protein accumulation by ELISA at 24 dpi. Three independent lines were tested for each construct.

Neither Columbia nor L*er Arabidopsis* plants are susceptible to the TEV CAA10 strain. The *iso4e rtm1* plants transformed with 35S:At.eIFiso4E constructs were challenged with TEV CAA10 but overexpression of At.eIFiso4E was not sufficient to trigger susceptibility in *Arabidopsis* (Figure 6). If this lack of susceptibility is linked to an active resistance, we would expect this mechanism to remain functional in the transgenic plants expressing *Ca.eIF4E1* alleles and the plants would remain resistant. On the contrary, if the resistance relies on an incompatibility mechanism, this resistance might be alleviated by overexpressing an eIF4E1 allele demonstrated to be required by TEV CAA10 in pepper. To test this, the *Arabidopsis* T2 lines expressing the pepper eIF4E1 alleles were challenged with TEV CAA10. Transgenic T2 plants overexpressing either Ca.eIF4E1-*pvr2*^+^ or Ca.eIF4E1-*pvr2*^*2*^ cDNAs were highly susceptible to TEV CAA10 (Figure 6). Therefore, expression of a heterologous susceptibility host factor is sufficient to create susceptibility in an otherwise incompatible accession. Interestingly, the transgenic *Arabidopsis* plants overexpressing Ca.eIF4E1-*pvr2*^*2*^ were resistant to TEV HAT but susceptible to TEV CAA10, mirroring precisely the resistance-breaking effect observed in the pepper/TEV pathosystem*.*

## Discussion

Viruses rely on host factors to complete their replicative cycle and successfully infect hosts. The plant initiation factors eIF4E and eIFiso4E and their respective partners eIF4G and eIFiso4G are examples of host factors that are required for potyviruses to infect plants. Recessive resistance (or impaired susceptibility) occurs mainly when the host factors are either absent or modified and cannot be used by the virus and this can explain some aspects of non-host resistance [29,30]. To infect multiple hosts, the pathogen must be able to adapt to different cellular mechanisms [31]. Here, we investigated how the TEV strain exploits different translation initiation factor isoforms to infect two plants from different dicotyledon genera, *Arabidopsis* and *Capsicum*.

After validating the complementation system, we were able to restore *Arabidopsis* susceptibility to TEV HAT by overexpressing the susceptible pepper *pvr2*^*+*^ allele encoding eIF4E1. The susceptible Ca.*eIF4E1-pvr2*^*+*^ allele is sufficient to replace the knocked-out *Arabidopsis eIFiso4E* and allow the virus to perform its infection cycle in *Arabidopsis*. The shift in the use of eIF4E isoform by TEV between pepper and *Arabidopsis* is surprising given that the Ca.eIF4E1 protein is much more similar to At.eIF4E1 than to At.eIFiso4E. Furthermore, regions I and II, which are crucial in determining susceptibility to viruses, are much more similar between Ca.eIF4E1 and At.eIF4E1 than between Ca.eIF4E1 and At.eIFiso4E. In pepper, resistance to the potyvirus *Pepper veinal mottle virus* and its close relative *Chilli veinal mottle virus* has been characterized as being digenic and to rely on both Ca.eIF4E1 and Ca.eIFiso4E [11,12], so a potyvirus can use both isoforms in the same plant. Similarly, overexpression of both eIF4E and eIFiso4E alleles from *Brassica rapa* in resistant *eifiso4e Arabidopsis* restores susceptibility to TuMV [32] showing that TuMV can use both isoforms as well. The TEV–4E system studied here is different in that the shift in the TEV requirement of the 4E isoform occurs between plant species and is highly specific. In other words, it is surprising that TEV HAT cannot use either At.eIF4E1 in *Arabidopsis* nor Ca.eIFiso4E in pepper. It is unlikely that this specificity arises from different expression patterns, notably because *At.eIF4E1* is also involved in susceptibility to ClYVV so it can be assumed that the broad expression pattern of *At.eIF4E1* expression makes it an available target for other potyviruses. Possibly, AA variations in regions I and II of AteIF4E1 make it incompatible with TEV, even though At.eIF4E1 does not share the polymorphisms of resistance allele *pvr2*^*2*^, V67E and L79R [17]. This hypothesis is consistent with the lack of interaction detected between At.eIF4E1 and the TEV VPg in yeast-two hybrid assays.

Gene redundancy can make it difficult to design experiments based on loss of biological function, and gain-of-function approaches offer an interesting alternative. For example, heterologous expression of four different *Brassica rapa eIF4E* and *eIFiso4e* genes in the resistant *eifiso4e Arabidopsis* mutant restored susceptibility to TuMV [32], although ectopic expression of the candidate genes can prove to be misleading [33]. We adopted a similar strategy to test translation initiation 4E genes isolated from the more distantly related *Arabidopsis* and pepper. Interestingly, the precise allele behaviour distinguishing between the different viral strains was maintained in *Arabidopsis*. Also, expressing either Ca.eIF4E1-*pvr2*^+^ or Ca.eIF4E1-*pvr2*^*2*^ alleles suppressed the incompatibility with the TEV CAA10 strain that normally cannot infect Col or L*er*. Similarly, *Carmovirus melon necrotic spot virus* (MNSV) Ma5 is able to multiply in *Nicotiana benthamiana* if a susceptible melon eIF4E is supplied [34]. Translation initiation factors are therefore a major determinant of susceptibility to positive-strand RNA viruses.

To set up the experimental system in *Arabidopsis*, we took advantage of the natural variation in resistance at the *RTM1* locus. By combining the *Ateifiso4e* mutation in Col accession with the natural *rtm1* allele from L*er*, it was possible to suppress the systemic dominant resistance to TEV in Col. This created a clear background in which to test transgenic overexpression of different eIF4E proteins and the effect on plant susceptibility. Variation in pepper e*IF4E1* genes was also exploited to compare the differential resistance to the two TEV strains. A large pool of eIF4E1 alleles has already been characterized in *Capsicum spp.*, [17,35,36] and the joint availability of next generation sequencing output and large germplasm collections is likely to enlarge this pool [37,38]. However, precisely dissecting the role of these alleles in resistance may also be hindered by the presence of interfering dominant resistances in the genetic background [35]. The genetic validation of such alleles may require difficult and time-consuming genetic studies if crosses between wild-relative species are incompatible. As well as the natural alleles available, allele-replacement technologies and mutagenesis approaches such as TILLING might offer better opportunities to generate tailor-made alleles in the near future. Testing alleles using an *Arabidopsis* susceptibility-complementation system, such as the one described here, could be a fast and cost-effective way to assess allele resistance to TEV in order to select the best ones for crop breeding strategies.

## Conclusions

Potyviruses can infect multiple hosts by relying on the host translation initiation factor 4E or its isoform iso4E. We show that *Arabidopsis thaliana* is a good heterologous system to assess whether 4E initiation factors from the crop plant pepper act as TEV resistant/susceptible alleles by overexpressing them in a resistant genetic background. *Arabidopsis* susceptibility to TEV that relies on eIFiso4E can be restored by the pepper eIF4E1 in a specific manner, showing that the idiosyncratic properties of the 4E and/or iso4E proteins create a major checkpoint allowing or not allowing the virus to infect different hosts. Moreover, this restoration of susceptibility is allele-specific, mimicking in *Arabidopsis* the behaviour of the eIF4E-*pvr2* allele in pepper. These results suggest that *Arabidopsis* could be a good model to assess new eIF4E alleles for resistance to TEV and may also be used to assess their durability.

## Methods

### Protein accession numbers and phylogeny

The following protein sequences were used for the phylogeny analysis with the accession numbers shown in brackets: Pd.eIF4E1 (JX137116) and Pd.eIFiso4E (JX137117) from *Prunus domestica*; Ps.eIF4E1 (AAR04332) and Ps.eIFiso4E (ABH09880) from *Pisum sativum*; Ls.eIF4E1 (AAP86602) and Ls.eIFiso4E (AAP86603) from *Lactuca sativa*; Ca.eIF4E1 (AAN74644) and Ca.eIFiso4E (AAY62607) from *Capsicum annuum*; At.eIF4E1 (NP_193538) and At.eIFiso4E (NP_198412) from *Arabidopsis thaliana*; and Sl.eIF4E1 (ABF83563) and Sl.eIFiso4E (ABV23495) from *Solanum lycopersicum*. The phylogenetic tree was generated using phylogeny.fr [39].

Protein sequences were aligned using MultiAlin (http://multalin.toulouse.inra.fr) and BoxShade (http://www.ch.embnet.org/software/BOX_form.html).

### Yeast two hybrid interaction assays

Protein-protein interaction was tested as previously described using the Matchmaker 3 yeast two-hybrid system (Clontech). The growth of yeast colonies containing both prey and bait vectors is shown as a control on Figure 2 on selective dropout medium lacking leucine and tryptophan (SD-LW) and interactions were selected on selective dropout medium lacking leucine, tryptophan and histidine (SD-LWH). Each combination was tested in triplicate. The TEV HAT VPg was fused to the binding domain (BD) of the GAL4 while the different eIF4E1 and eIFiso4E were fused to the activation domain (AD). All plasmids have been described previously [17,20].

### Plasmid constructions

All plasmids and oligonucleotides used in this study are listed in Additional file 1: Table S1 and Additional file 2: Table S2, respectively. Entry clones were prepared by RT-PCR amplification introducing the attB1/attB2 Gateway recombination sequences followed by BP clonase recombination into the pDONR207 vector (Invitrogen). All clones were checked by sequencing before further use. Other clones were obtained by LR clonase recombination reactions in the destination vector pMDC32 for CaMV 35S-driven overexpression [40].

### Plant materials and plant transformation

*Arabidopsis thaliana* Columbia 0 (Col) plants were used as the wild-type control and the Landsberg *erecta* (L*er*) accession was used for its *rtm1* mutant allele [28]. The homozygous *Ateifiso4e* KO allele caused by insertion of a dSpm element has been described before [6]. Plants were grown at 18 to 20°C, with 16-h light (100 μmol photons m^-2^ s^-1^ of fluorescent light) and 8-h dark cycles. For virus tests, plants were growth in the same conditions but in short days (8 h of light).

For genetic crosses, immature flowers were emasculated and manually cross-pollinated. All binary vectors were transformed into *Ateifiso4e rtm1 Arabidopsis* plants using the floral dip method [41]. All T1 and T2 plants were selected on germination medium plates supplemented with 15 mg/L hygromycin B. About 10 independent T1 plants were selected for each construct and allowed to self. The presence of the transgene in T2 plants was shown by plant resistance to hygromycin and PCR genotyping. The *eifiso4e rtm1* background was also confirmed by genotyping (Additional file 3: Figure S1). Control plants expressing the GUS reporter gene were checked by GUS staining (data not shown).

### Virus inoculation and detection by ELISA

The TuMV CDN1 strain [20] and both TEV HAT and CAA10 strains [17] were propagated on turnip (*Brassica rapa*) and *Nicotiana benthamiana* cv Xanthi, respectively. Viruses were inoculated on 1-month-old *Arabidopsis* and TuMV and TEV viral accumulation was assayed after 24 days by ELISA using respectively AntiPoty (Agdia) and AntiTEV (Sediag) antisera and detection sets. All results presented are mean values from 6 independent plants per genotype and error bars represent standard errors. The threshold for susceptibility is represented by a line on each graph and refers to an absorbance value at 405 nm in ELISA equal to three times the mean value for healthy controls. Both Yolo Wonder and Florida VR2 pepper accessions were used as controls in all viral infections throughout this study (Additional file 4: Figure S2).

### Plant genotyping and RT-PCR

The *Ateifiso4e* mutant allele is caused by the insertion of a defective *dSpm* element into the second exon of *AteIFiso4E* (At5g35620). The wild-type allele was PCR-genotyped on genomic DNA with primers Z2835 and Z2836 and the mutant allele was genotyped using primers Z2835 and Z524, an oligonucleotide that hybridises at the 3’end of the *dSpm* element. The *rtm1* allele was genotyped with a CAPS marker as follows. A 340-bp DNA fragment covering the *RTM1* locus was amplified with primers Z2322F and Z2323F and digested with restriction enzyme HinfI. The fragments resulting from the Col *RTM1* allele and the L*er rtm1* alleles resulted in main bands of 260 bp and 298 bp, respectively.

### Antibodies and western blot

The complete AteIFiso4E cDNA sequence was cloned into the expression vector pET15b (Novagen). Recombinant His-tagged AteIFiso4E protein was produced, purified and used to produce polyclonal antibodies in rabbits (New Zealand White, SPF) by Proteogenix (Oberhausbergen, France). The resulting serums were purified against the His-tagged AteIFiso4E protein by affinity purification.

For western blot analysis, total proteins were extracted from 1-month-old leaves in Laemmli buffer. Equal amounts of protein extracts were electrophoresed on an SDS-polyacrylamide gel and blotted onto Hybond ECL nitrocellulose membranes (GE Healthcare, Buckinghamshire, UK). The anti-AteIFiso4E serum was diluted at 1/2000 and combined with a secondary goat anti-HRP-labelled anti-rabbit serum (Sigma-Aldrich) diluted at 1/5000. As loading control, monoclonal anti-plant actin antibodies (1/2000 dilution) (Sigma-Aldrich) were used with HRP-labelled rabbit anti-mouse serum (1/2000 dilution) (Sigma-Aldrich). HRP activity was detected using the LumiGLO Reserve chemiluminescent substrate kit (KPL, Les Ulis, France) and X-OMAT LS films (Kodak).

### Reverse transcription analysis

Total RNA was extracted using TRI-Reagent (Sigma-Aldrich) from 1-month-old leaves. Contaminating DNA was removed by DNAse I treatment. RT-PCR was performed with AMV reverse transcriptase (Promega) on 1 μg of total RNA according to the supplier’s instructions. *ADENINE PHOSPHORIBOSYL TRANSFERASE 1* (*APT1*, At1g27450) was used as a constitutive control. *C. annuum eIF4E1* and *APT1* cDNA were amplified using Z3221-Z3222 and Z1734-Z1735 primer pairs, respectively.

## Competing interests

The authors declare that they have no competing interest.

## Authors’ contributions

JLG designed the experiments. JE, AMa, CCallot, AMo and JLG carried out the experiments. SL contributed new reagents. CCaranta and JLG wrote the manuscript. All authors read and approved the final manuscript.

## Supplementary Material

Additional file 1: Table S1 List of oligonucleotides used in this study. Click here for file

Additional file 2: Table S2 List of plasmids used in this study. Click here for file

Additional file 3: Figure S1 Genotyping of transgenic Arabidopsis T2 plants. For each construct, results from 3 independent T2 are shown. **A**, Genotyping of the *iso4e rtm1* genetic background of the T2 transgenic plants (see Methods). **B**, Genotyping of the inserted T-DNA allowing the overexpression of *At.eIFiso4E*, Ca.*eIF4E1-pvr2*^*+*^ and Ca.*eIF4E1-pvr2*^*2*^, respectively. Click here for file

Additional file 4: Figure S2 Control test of TEV susceptibility on Capsicum annuum Yolo Wonder and Florida VR2 accessions. Plants were mechanically inoculated with TEV HAT or TEV CAA10 at the cotyledon stage and assayed for viral coat accumulation by ELISA at 24 dpi. Click here for file
