# Supplementary material for: Specific requirement for translation initiation factor 4E or its isoform drives plant host susceptibility to Tobacco etch virus
Source: BMC Plant Biol. 2014 Mar 19;14:67. doi: 10.1186/1471-2229-14-67 (PMC3999954; doi:10.1186/1471-2229-14-67)
Supplement: Additional file 3: Figure S1 — Genotyping of transgenic Arabidopsis T2 plants. For each construct, results from 3 independent T2 are shown. A, Genotyping of the iso4e rtm1 genetic background of the T2 transgenic plants (see Methods). B, Genotyping of the inserted T-DNA allowing the overexpression of At.eIFiso4E, Ca.eIF4E1-pvr2+ and Ca.eIF4E1-pvr22, respectively. [file 1471-2229-14-67-S3.ppt]

## Slide 1
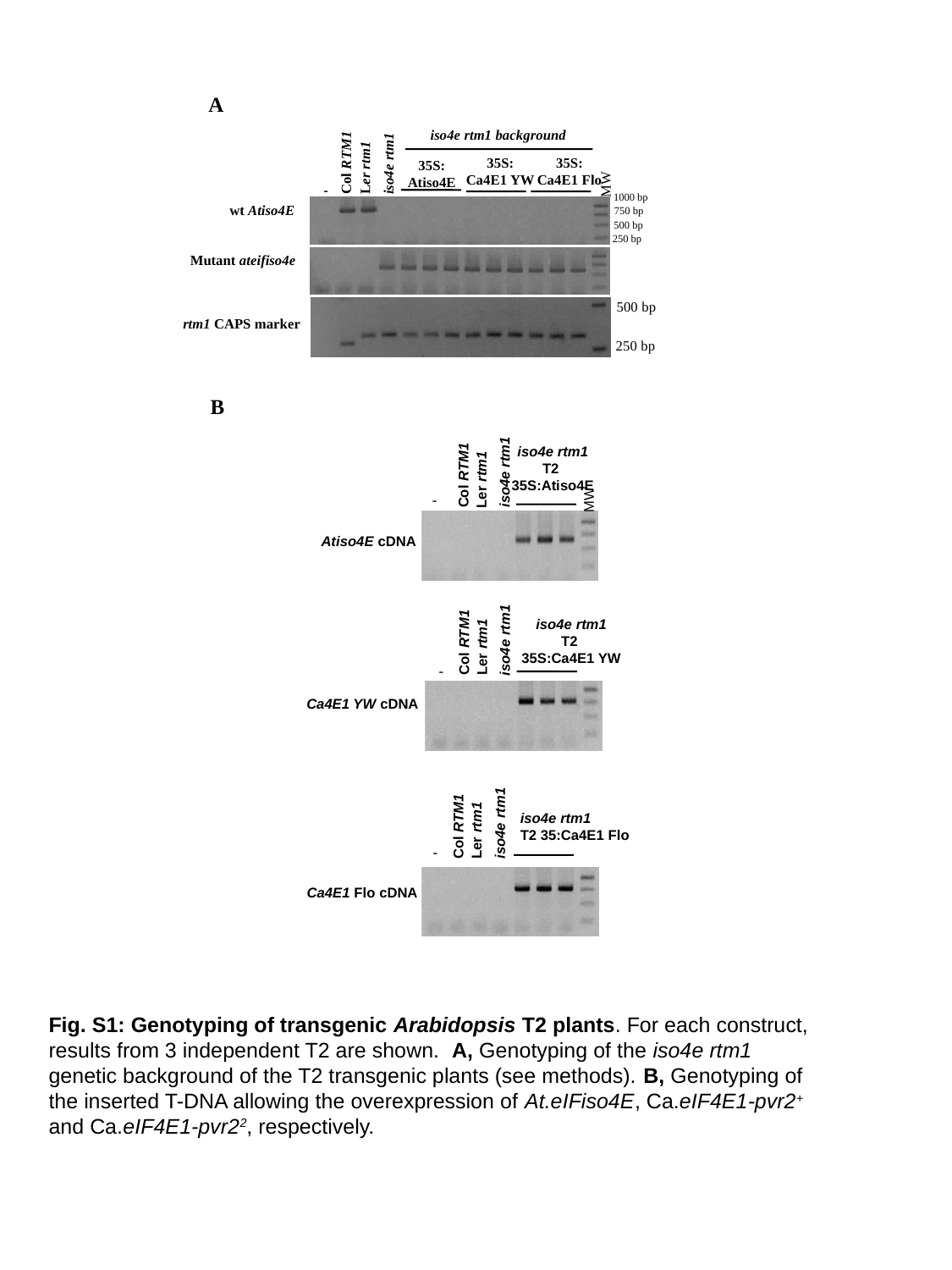

A
iso4e rtm1 background
35S:
Ca4E1 Flo
Col RTM1
35S:
Ca4E1 YW
iso4e rtm1
35S:
Atiso4E
Ler rtm1
MW
-
1000 bp
wt Atiso4E
750 bp
500 bp
250 bp
Mutant ateifiso4e
500 bp
rtm1 CAPS marker
250 bp
B
iso4e rtm1
T2
35S:Atiso4E
iso4e rtm1
Col RTM1
Ler rtm1
-
MW
Atiso4E cDNA
iso4e rtm1
T2
35S:Ca4E1 YW
iso4e rtm1
Col RTM1
Ler rtm1
-
Ca4E1 YW cDNA
iso4e rtm1
T2 35:Ca4E1 Flo
iso4e rtm1
Col RTM1
Ler rtm1
-
Ca4E1 Flo cDNA
Fig. S1: Genotyping of transgenic Arabidopsis T2 plants. For each construct, results from 3 independent T2 are shown. A, Genotyping of the iso4e rtm1 genetic background of the T2 transgenic plants (see methods). B, Genotyping of the inserted T-DNA allowing the overexpression of At.eIFiso4E, Ca.eIF4E1-pvr2+ and Ca.eIF4E1-pvr22, respectively.
