# Supplementary material for: Specific requirement for translation initiation factor 4E or its isoform drives plant host susceptibility to Tobacco etch virus
Source: BMC Plant Biol. 2014 Mar 19;14:67. doi: 10.1186/1471-2229-14-67 (PMC3999954; doi:10.1186/1471-2229-14-67)
Supplement: Additional file 4: Figure S2 — Control test of TEV susceptibility on Capsicum annuum Yolo Wonder and Florida VR2 accessions. Plants were mechanically inoculated with TEV HAT or TEV CAA10 at the cotyledon stage and assayed for viral coat accumulation by ELISA at 24 dpi. [file 1471-2229-14-67-S4.ppt]

## Slide 1
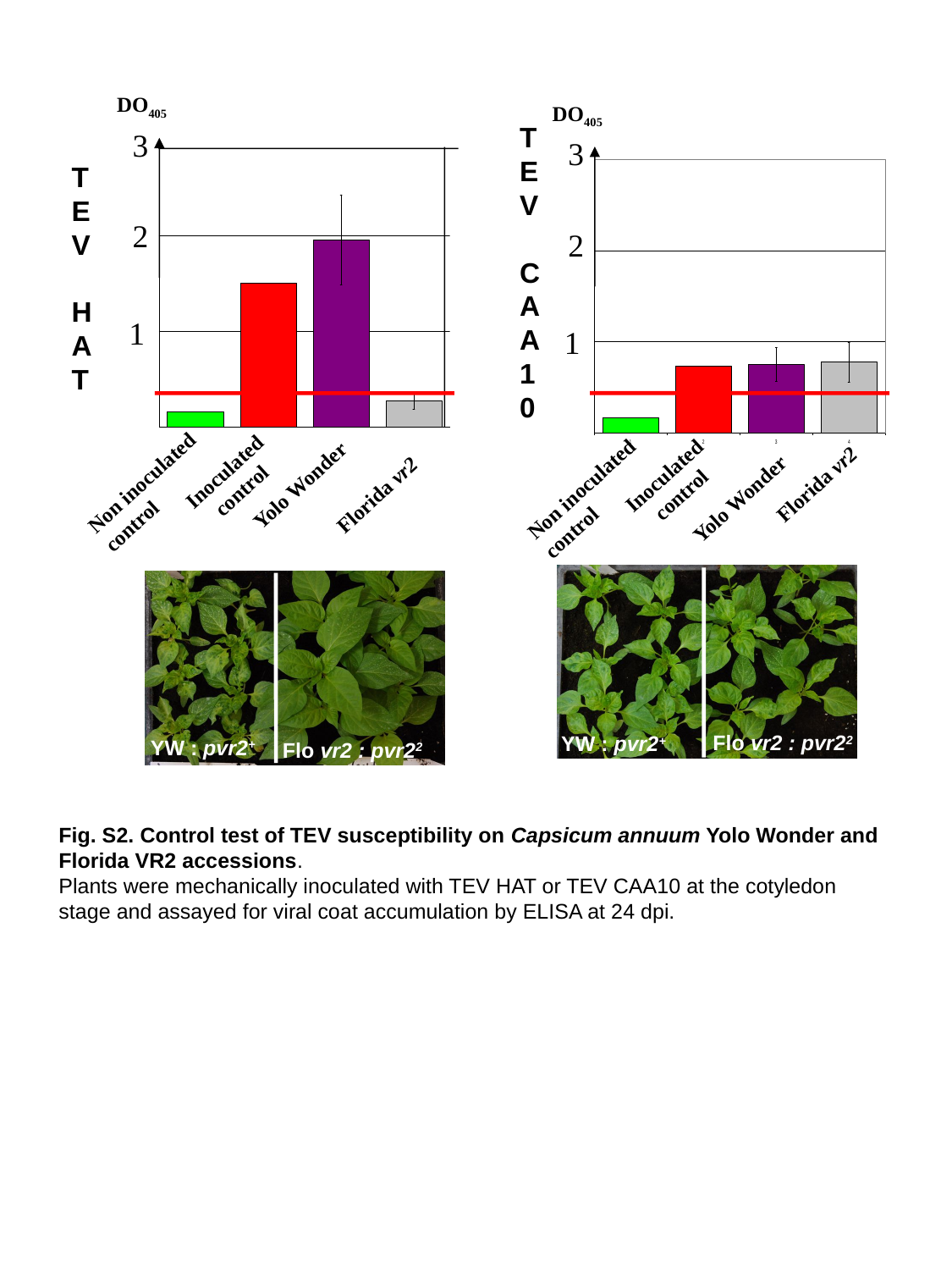

DO405
3
TEV
 HAT
2
1
Inoculated control
Yolo Wonder
Florida vr2
YW : pvr2+
Flo vr2 : pvr22
Non inoculated control
DO405
TEV
 CAA10
3
2
1
Inoculated control
Florida vr2
Non inoculated control
Yolo Wonder
Flo vr2 : pvr22
YW : pvr2+
Fig. S2. Control test of TEV susceptibility on Capsicum annuum Yolo Wonder and Florida VR2 accessions.
Plants were mechanically inoculated with TEV HAT or TEV CAA10 at the cotyledon stage and assayed for viral coat accumulation by ELISA at 24 dpi.
